# 1RS arm of *Secale cereanum* ‘Kriszta’ confers resistance to stripe rust, improved yield components and high arabinoxylan content in wheat

**DOI:** 10.1038/s41598-020-58419-3

**Published:** 2020-02-04

**Authors:** Éva Szakács, Kitti Szőke-Pázsi, Balázs Kalapos, Annamária Schneider, László Ivanizs, Marianna Rakszegi, Gyula Vida, István Molnár, Márta Molnár-Láng

**Affiliations:** 1 Agricultural Institute, Centre for Agricultural Research, Martonvásár, H-2462 Hungary; 2 National Food Chain Safety Office, Budapest, 1024 Hungary; 3 Institute of Experimental Botany, Centre of the Region Haná for Biotechnological and Agricultural Research, Šlechtitelů 31, CZ-, 78371 Olomouc, Czech Republic

**Keywords:** Plant breeding, Plant genetics, Biotic, Plant sciences, Agricultural genetics

## Abstract

Wheat-rye T1BL.1RS translocation is widespread worldwide as the genes on 1RS arm have positive effect on stress resistance, grain yield and adaptation ability of wheat. Nowadays, the T1BL.1RS wheat cultivars have become susceptible to rust diseases because of the monophyletic (‘Petkus’) origin of 1RS. Here we report and discuss the production and detailed investigation of a new T1BL.1RS translocation line carrying 1RS with widened genetic base originating from *Secale cereanum*. Line ‘179’ exhibited improved spike morphology traits, resistance against stripe rust and leaf rust, as well as higher tillering capacity, fertility and dietary fiber (arabynoxylan) content than the parental wheat genotype. Comparative analyses based on molecular cytogenetic methods and molecular (SSR and DArTseq) makers indicate that the 1RS arm of line ‘179’ is a recombinant of *S. cereale* and *S. strictum* homologues, and approximately 16% of its loci were different from that of ‘Petkus’ origin. 162 (69.5%) 1RS-specific markers were associated with genes, including 10 markers with putative disease resistance functions and LRR domains found on the subtelomeric or pericentromeric regions of 1RS. Line ‘179’ will facilitate the map-based cloning of the resistance genes, and it can contribute to healthy eating and a more cost-efficient wheat production.

## Introduction

Interspecific hybridization is one of the most promising way to improve the genetic diversity of bread wheat^1^. The most widely known wheat-alien introgression has been the spontaneous translocation T1BL.1RS that is traced back to the cross of wheat ‘Criewener 104’ × rye ‘Petkus Roggen’ made in Germany between 1920 and 1930^2^. Its effect on morphology and baking quality of bread wheat has been investigated in numerous studies. The positive effect of this translocation on grain yield of hexaploid wheat^3,4^ and durum wheat^5,6^ is commonly accepted, though in some cases no such effect was shown^7,8^. Association between the presence of 1RS and increase in root biomass, water use efficiency as well as harvest index was also found in glasshouse and field experiments^9,10^.

1RS arm also contains genes (*Pm8, Lr26, Sr31* and *Yr9*) providing resistance against powdery mildew (*Erysiphe graminis* f. sp. *tritici*), leaf rust (*Puccinia triticina*), stem rust (*Puccinia graminis* f. sp. *tritici*), and stripe or yellow rust (*Puccinia striiformis* f. sp. *tritici*), respectively^11,12^, a reason that led to the widespread utilization of the T1BL.1RS translocation in wheat improvement. However, the resistance genes *Lr26*, *Yr9* and *Pm8* are no longer effective against new virulent biotypes of the pathogens in Europe^13^. Virulence to the *Sr31* resistance gene has also been reported from Uganda^14^, and from Kenya, Ethiopia, Sudan, and Iran^15^. Spread of the *Sr31-*virulent pathotype in countries where wheats carrying the T1BL.1RS chromosome are cultivated can cause serious problems for agriculture as the genetic vulnerability of T1BL.1RS cultivars is the consequence of the lack of allelic variation on the ‘Petkus’-derived 1RS arm^4^. The importance of widening the genetic base of cultivated wheat by introducing new rye genetic resources into resistance breeding programmes has grown^16,17^.

The diploid wild mountain rye *Secale strictum* (C. Presl) C. Presl (syn. *Secale montanum* Guss.) is a close relative of *S. cereale*^18^ and considered as rich source of genes and alleles for rye and wheat improvement. Compared to cultivated rye, it has strong tillering ability, tolerance to frost and drought^19^, heavy metal^20,21^ and aluminium-stresses^22^, as well as resistance to biotic stresses such as rust diseases and Russian wheat aphids^23^.

In Hungary, artificial hybrids between *S. cereale* cv. Várda and *S. strictum* ssp. *anatolicum* were developed in the 1960’s in order to combine favourable traits of these ryes^24^. The perennial rye (*S. cereanum*) variety Kriszta bred from these hybrids by Kruppa^25^ is resistant to leaf rust, stem rust, stripe (yellow) rust and powdery mildew, its winter hardiness and drought tolerance are very good, and has good nutritional parameters including high dietary fiber (DF) content. Non-starch cell wall polysaccharides, such as arabinoxylan, which is the main component of DFs in wheat and rye, provide many health benefits as their insoluble forms decrease the absorption of carcinogens, while soluble DFs reduce the probability of coronary heart disease and type II diabetes (for review see^26^). Because of wheat’s central role in human nutrition, development of varieties with increased DF content is an outstanding goal in breeding programs.

Bread making quality, another important aspect of wheat breeding, relates dominantly with the gluten storage proteins, as monomeric gliadins (Gli) determine dough extensibility, while polymeric glutenins (Glu) its elasticity. The high ratio of unextractable polymeric glutenin proteins (UPP) is often associated with good quality^27,28^.

Incorporation of suitable alleles from rye is a viable way to increase DF content of wheat, but the chromosome mediated gene transfer may also modify the composition of storage proteins. Therefore, a further objectives of this study was to determine whether the ‘Kriszta’ 1RS chromosome arm affects the DF and gluten storage protein content and composition of the parental wheat Mv9kr1.

Conventionally, rye chromatin in wheat genetic background can be detected and identified by using genomic and fluorescence *in situ* hybridization (GISH and FISH)^29,30^. The satellite repeat pSc119.2 isolated from rye and producing chromosome-specific hybridization pattern in many species of *Triticeae* is generally used as DNA probe for the karyotype analysis of wheat and rye, and their hybrid progenies^31^. In a comparative analysis of *S. cereale* and various wild *Secale* species using six repetitive DNA sequences including pSc119.2, Cuadrado and Jouve (2002) concluded that the species studied are unique in the chromosomal distribution of the sequences analyzed^32^. Accordingly, the hybridization pattern of pSc119.2 can be considered appropriate cytogenetic marker to differentiate between *S. cereale* and *S. strictum* chromosomes. PCR-based SSR molecular markers are suitable to detect rye chromatin introgressed into wheat, but because of the low throughput, the high resolution analysis of wheat-rye introgression lines is time consuming by this marker system^33,34^.

The first, high-throughput genotyping technology developed for rye was the Diversity Arrays Technology (DArT), which was a hybridization-based microarray platform^35^. DArT markers are DNA fragments obtained by the genome complexity reduction method including the digestion of genomic DNA by *Pst*I/*Taq*I endonucleases, ligation of the genomic fragments to a *Pst*I adapter, amplification using a primer complementer with the adaptor sequence and transformation into *E. coli*. Inserts of individual colonies were then amplified, printed on microarrays and used for genotyping^36^. The DArTseq platform combines the genome complexity reduction method of DArT^37,38^ and the genotyping-by-sequencing technology^39,40^. It provides two types of markers: Silico-DArT and SNP-DArT markers (https://www.diversityarrays.com/). Silico-DArT markers are dominant and reflect the presence/absence variation of the genomic fragment, while the SNP-DArT markers show the nucleotid polymorphism within the genomic fragment. DArTseq technology provides several thousands of high-quality molecular markers in a rapid and cost-effective way for genome profiling without the need of prior sequence information. Due to the use of methylation-sensitive restriction endonucleases (*Pst*I) in combination with frequently cutting enzymes (*Taq*I), the heavily methylated repetitive DNA fraction can be excluded from a defined DNA sample, consequently, DArTseq markers represent low-copy sequences (gene-rich regions) in high percent. Using polymorphic DArTseq markers, Milczarski *et al*. (2016)^41^ extended the high-density map of rye, and were able to localize the *Rfc1* male fertility restorer gene on the long arm of the 4R chromosome, Al-Beyroutiová *et al*. (2016)^42^ evaluated genetic diversity and phylogenetic relationships between annual, perennial and semi-perennial *Secale* species, and Targońska-Karasek *et al*. (2017)^43^ assessed genetic diversity in rye inbred lines. Rakoczy-Trojanowska *et al*. (2017)^44^ identified SNPs associated with brown rust (leaf rust) resistance, α-amylase activity and pre-harvest sprouting in rye breeding materials.

The main goal of the present study was to widen the genetic diversity of the 1RS chromosome arm introgressed into wheat by producing a new, disease resistant T1BL.1RS translocation line deriving from wheat (Mv9kr1) x *Secale cereanum* (‘Kriszta’) hybrids. The main objectives (summarized in Table 1) were to investigate how the new T1BL.1RS chromosome altered the infection responses to stripe- and leaf rust and powdery mildew, the morphological and yield component traits and the dietary fiber content of the recipient wheat genotype. As omega-secalins (ω-secalins) encoded by the *Sec-1* locus on the chromosome arm 1RS may influence the bread making quality, the ω-secalin subunit and storage protein compositions of the new introgression line were also compared with those of the parental wheat genotype.

**Table 1:** Summary of the aims of this study, and the methods and genotypes used in the experiments.

Aim	Method	Genotype
Assessment of infection responses to natural stripe rust infections under field conditions (2014–2016), in order to compare the resistance of the lines carrying 1RS of different origin.	Visual observation and scoring according to the modified Cobb scale (percentages of infected leaf area)	**Mv9kr1** (parent of ‘179’), **‘Kriszta’** (parent of ‘179’), **‘179’, ‘C5’, ‘D5’** (T1BL.1RS wheat lines), **DA1R** (Mv9kr1/‘Kriszta’ 1R addition line), ‘**Mv Magdaléna’** (T1BL.1RS - ‘Petkus’)
Comparision of infection responses to leaf rust and powdery mildew under greenhouse conditions.	Artificial inoculation	**Mv9kr1, ‘Kriszta’, ‘179’, ‘Mv Magdaléna’**
Determination of morphological changes caused by the presence of ‘Kriszta’ 1RS (and/or lack of 1BS) in the Mv9kr1 genetic background.	Measurements both in field and after harvest	**Mv9kr1, ‘Kriszta’, ‘179’**
Detection of putative cytomolecular differences between 1RS chromosme arm of the stripe rust-susceptible ‘Mv Magdaléna’ (‘Petkus’ origin) and that of the resistant wheat line ‘179’.	Fluorescent *in situ* hybridization with repetitive DNA probe pSc179.2	**Várda** (*S. cereale*, parent of ‘Kriszta’), ***S. strictum*** (parent of ‘Kriszta’), **‘R797’**, **‘Kriszta’** (*S. cereanum* parent of ‘179’), **‘179’, ‘Mv Magdaléna’**
Detection of changes in the storage protein composition and dietary fiber content of the line ‘179’ caused by the presence of ‘Kriszta’ 1RS arm in the Mv9kr1 genetic background.	Kjeldahl-method SE-HPLC RP-HPLC Colorimetry	**Mv9kr1, ‘Kriszta’, ‘179’**
Detection of putative differences in the ω-secalin subunit composition encoded by the *Sec-1* locus on 1RS arms of different origin (‘Petkus’ vs. ‘Kriszta’).	Acid-polyacrylamid gel electrophoresis	**Mv9kr1, ‘Kriszta’, ‘179’, ‘Mv Magdaléna’, Chinese Spring** (as a reference)
Detection of putative differences at the DNA level between 1RS arms of ‘Petkus’ and ‘Kriszta’ origin, and selection of ‘Kriszta’ 1RS-specific markers for future use in development of other 1RS introgression lines.	SSR and ISBP marker analyses	**Mv9kr1, ‘Kriszta’, ‘179’, ‘Mv Magdaléna’**
Assessment of allelic variations between stripe rust-resistant and -sensitive T1RS.1BL lines.	DArTseq platform	**Mv9kr1** (susceptible), **‘179’, ‘C5’, ‘D5’** (resistant), **‘Mv Magdaléna’** (susceptible), **DA1R** (susceptible)

A further aim was to compare the molecular structure of the ‘Petkus’- and ‘Kriszta’-originated 1RS arms and find allelic variations between stripe rust-resistant and -susceptible wheat genotypes. For the reasons that the T1BL.1RS ‘Mv Magdaléna’ carries 1RS of ‘Petkus’ rye^16^ and is susceptible for stripe rust, we used this wheat variety for the comparative molecular cytogenetic, SSR and DArTseq marker analyses. The stripe rust-susceptible Mv9kr1-‘Kriszta’ 1R disomic addition line (DA1R)^45^ was also involved in this study.

## Results

### Production and selection of lines ‘179’, ‘C5’ and ‘D5’ by resistance to stripe rust

The hexaploid wheat Mv9kr1 x *Secale cereanum* cv. Kriszta hybrid plants were backcrossed and selfed several times (see Supplementary Fig. S1). Selection of disease resistant plants was carried out in the field over ten years from 2005 to 2014.

**Figure 1:**
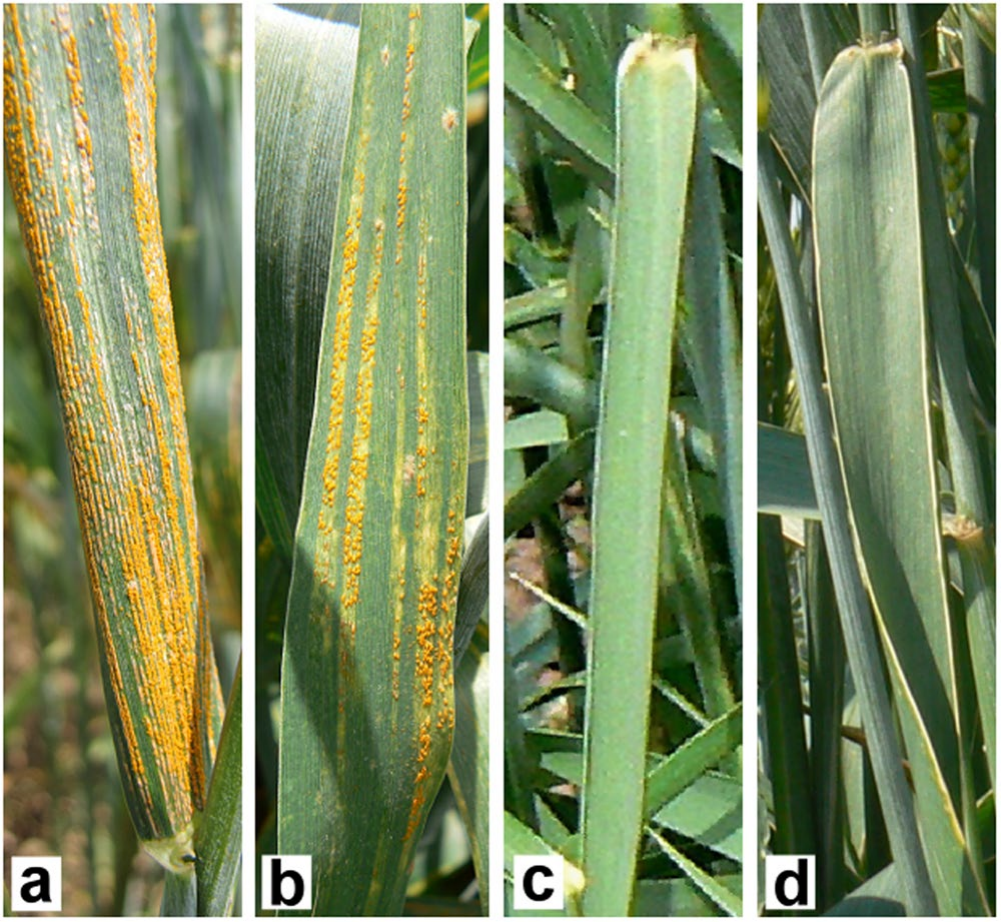
Stripe rust infection in the high-input PB nursery (Martonvásár, 2014). **(a)** severely infected leaf of the parental wheat line Mv9kr1; **(b)** infected leaf of the T1BL.1RS cultivar Mv Magdaléna; **(c,d)** leaves without any symptome of the parental perennial rye *S. cereanum* cultivar Kriszta and the Mv9kr1-‘Kriszta’ (wheat-*S. cereanum*) line ‘179’, respectively.

BC_2_F_8_ generation of these genotypes were sown in the nursery and their adult plant resistance to natural infection by stripe rust was recorded in three consecutive years (2014, 2015 and 2016). The susceptible parental wheat line Mv9kr1 and the Mv9kr1-‘Kriszta’ disomic addition line DA1R suffered from serious infection (scores 80 to 100 on the modified Cobb scale) and ‘Mv Magdaléna’ (carrying ‘Petkus’ 1RS) was also susceptible (scores 60 to 80), while the lines ‘179’, ‘C5’ and ‘D5’ showing no visible symptoms (score 0) proved to be resistant each year (Fig. 1, Supplementary Table S1).

**Figure 2:**
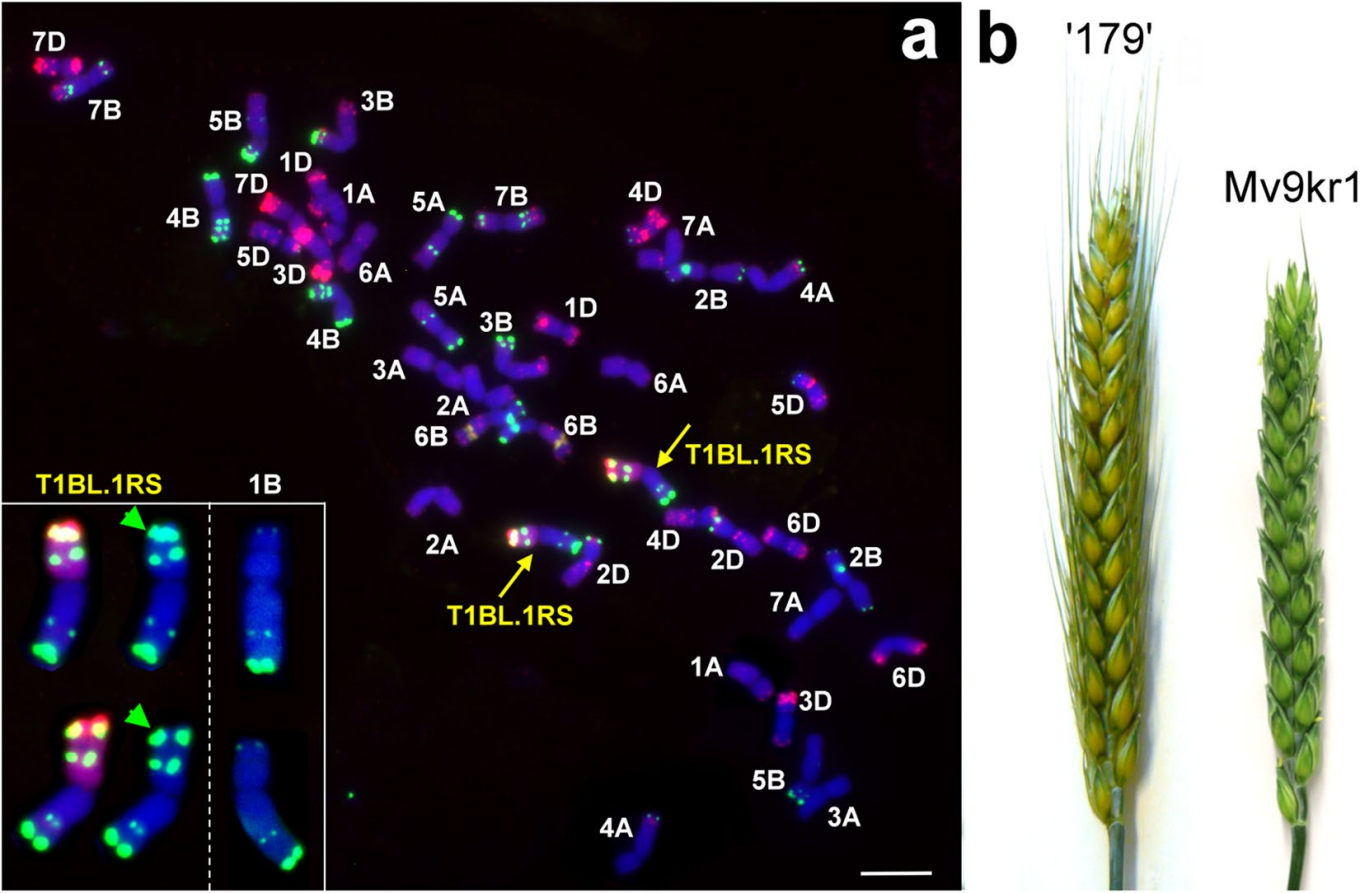
Cytomolecular identificaion of the line ‘179’. (**a**) Simultaneous FISH with DNA sequences (pSc119.2 - green, Afa-family - red, pTa71 - orange) and GISH on mitotic chromosome spread of the Mv9kr1-‘Kriszta’ (wheat-*S. cereanum*) line ‘179’. T1BL.1RS chromosomes (arrowed) together with a pair of ‘Mv9kr1 1B chromosomes are enlarged at the bottom-left corner. Double pSc119.2 pattern specific for 1RS of line ‘179’ is indicated by green arrowheads. Red GISH signal is visibile on the 1RS chromosome arm. Scale bar: 10 µm. (**b**) Spike morphology of the wheat lines ‘179’ and Mv9kr1.

Lines ‘179’, ‘C5’ and ‘D5’ were classified as T1BL.1RS translocations, as the hybridization pattern of probe pSc119.2 was typical for the rye chromosome arm 1RS (strong signal on the satellite and another one proximal from the secondary constriction) and for the long arm of 1B (Fig. 2a) in all the three genotypes.

As ‘179’ was the first and from the breeder’s point of view a promising (uniform) genotype we selected, in the subsequent parts of our study we focus on the presentation of this line. Genotypes ‘C’ and ‘D’ were involved only in studies on allelic differences between the ‘Petkus’- and ‘Kriszta’-originated 1RS chromosome arms.

### Analysis of morphological traits

The 1BL.1RS translocation significantly modified the morphology of the wheat parent. Compared to Mv9kr1, line ‘179’ possesses awned and longer spikes with higher number of spikelets (Fig. 2b, Table 2, Supplementary Fig. S2). The improved spike architecture of ‘179’, due to the higher number of tillers, resulted in an approximately 50% increase in the number of seeds per main spike and an increase of about 80% in the number of seeds per plant.

**Table 2:** Morphological features of the Mv9kr1-‘Kriszta’ line ‘179’ and the parental lines *S. cereanum* ‘Kriszta’ and hexaploid wheat genotype Mv9kr1 in 2015 and 2019. Values are the means ± standard deviations of 10 measurements and were compared to those of the parental wheat line Mv9kr1. TKW: thousand kernel weight, PB: pre-breeding, LI: low-input.

Morphological trait	Nursery	Genotype		
		‘Kriszta’	Mv9kr1	Line ‘179’
Plant height (cm)	2015 PB	139.2 ± 12.9	68.9 ± 2.3	81.0 ± 4.4^**††^
	2019 PB	171.2 ± 1.9	88.5 ± 1.8	93.5 ± 1.9^**††^
	2019 LI	179.1 ± 4.5	87.4 ± 1.5	111.8 ± 1.4^**††^
Spikes per plant	2015 PB	12.9 ± 1.9	5.6 ± 2.2	8.0 ± 1.4^*††^
	2019 PB	16.7 ± 1.0	6.9 ± 1.0	10.3 ± 0.8^**††^
	2019 LI	16.1 ± 1.6	6.80 ± 0.8	11.2 ± 1.3^**††^
Length of the main spike (cm)	2015 PB	16.2 ± 1.8	8.3 ± 0.5	9.1 ± 0.4^**††^
	2019 PB	14.1 ± 0.7	10.3 ± 0.5	11.7 ± 0.9^**††^
	2019 LI	15.7 ± 1.3	10.7 ± 0.6	12.2 ± 0.6^**††^
Spikelets per main spike	2015 PB	40.6 ± 4.9	19.9 ± 1.5	24.0 ± 1.3^**††^
	2019 PB	36.8 ± 2.7	21.0 ± 1.3	25.1 ± 1.0^**††^
	2019 LI	42.6 ± 3.2	22.5 ± 1.3	26.6 ± 1.3^**††^
Seeds per main spike	2015 PB	66.2 ± 12.9	50.1 ± 4.0	74.8 ± 4.2^**††^
	2019 PB	66.7 ± 2.7	52.3 ± 3.5	70.9 ± 6.0^**††^
	2019 LI	70.5 ± 5.0	53.2 ± 2.0	77.7 ± 5.1^**††^
Seeds per plant	2015 PB	682.2 ± 64.3	230.8 ± 37.7	415.2 ± 52.4^*††^
	2019 PB	830.2 ± 57.7	346.0 ± 52.5	595.7 ± 64.3^**††^
	2019 LI	881.0 ± 45.7	361.6 ± 14.6	677.1 ± 62.8^**††^
TKW (g)	2015 PB	16.86 ± 0.3	33.78 ± 1.0	29.20 ± 0.7^**††^
	2019 PB	14.8 ± 0.5	35.4 ± 1.8	27.4 ± 1.4^**††^
	2019 LI	18.4 ± 2.0	37.2 ± 3.2	29.3 ± 0.9^**††^

*Significantly different from Mv9kr1 at the P < 0.05 level.

**Significantly different from Mv9kr1 at the P < 0.01 level.

^††^Significantly different from ‘Kriszta’ at the P < 0.01 level.

### Artificial disease resistance tests

**Figure 3:**
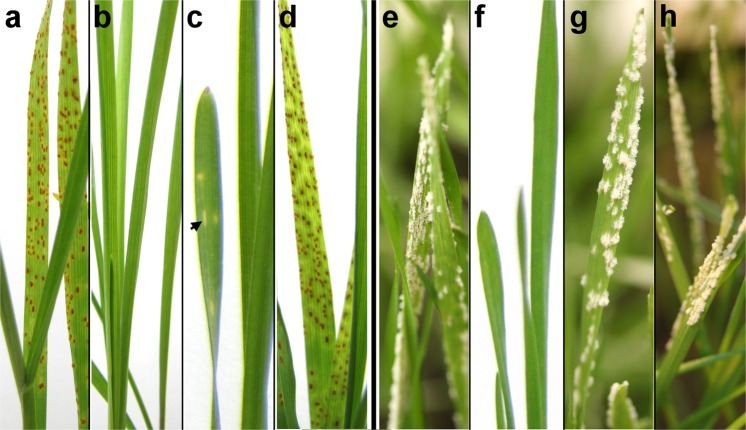
Artificial leaf rust (*Puccinia triticina*) (**a–d)** and powdery mildew (*Blumeria graminis* f.sp. *tritici*; isolate LH07–14) (**e–h)** resistance tests. (**a** and **e**) Infected leaves of the parental wheat genotype Mv9kr1. (**b** and **f**) Resistant leaves of the parental perennial rye ‘Kriszta’. (**c**) Seedling of line ‘179’ showing hypersensitive response to the infection. One of the yellow necrotic spots is indicated with arrowhead. (**g**) Infected leaf of the T1BL.1RS traslocation line ‘179’ susceptible to powdery mildew. (**d** and **h**) Leaves of the susceptible ‘Mv Magdaléna’ (carrying 1RS from ‘Petkus’ rye). Experiments were carried out on 10-day-old seedlings under greenhouse conditions.

As the rye chromosome arm 1RS harbours resistance genes against leaf rust (*Puccinia triticina*) and powdery mildew (*Blumeria graminis f. sp. tritici*), artificial resistance tests were performed to assess infection response of ‘179’ to these pathogens (Table 1). Infections were carried out using pathotypes with known virulence/avirulence formula (Supplementary Table S2). Leaf rust infenction caused hypersensitive lesions (necrotic spots) without uredia production on the leaves of line ‘179’ (carrying ‘Kriszta’ 1RS arm) indicating its resistance, while the parental wheat genotype Mv9kr1 and the variety Mv Magdaléna (carrying ‘Petkus’ 1RS) were seriously infected (Fig. 3a–d and Supplementary Table S3). ‘Kriszta’ exhibited immunity to the infection. During the powdery mildew infection test, ‘Mv Magdaléna’ and Mv9kr1 showed severe symptoms, while ‘Kriszta’ was immune. Line ‘179’, in contrast to our expectations, was infected with both isolates to the same extent as the susceptible Mv9kr1 (Fig. 3e–h, Supplementary Table S3).

### Quality measurements

Knowing that rye has higher dietary fiber content than wheat, we also investigated the effect of 1RS arm on the arabinoxylan (AX, a major cell wall polysaccharide) content and the protein composition of the wholemeal in ‘179’ together with the wheat and rye parents (Table 1). Line ‘179’ exhibited significantly higher (increased by 40.8%) total arabinoxylan (TOT-AX) content than the parental wheat Mv9kr1 reaching the level of the parental rye variety ‘Kriszta’ (Table 3). The increase in the level of water extractable arabinoxylans (WE-AX) was even higher (66.8%) in ‘179’ relative to the wheat parent.

**Table 3:** Quality properties of the parental perennial rye (*S. cereanum*) ‘Kriszta’, the wheat line Mv9kr1, and the Mv9kr1-‘Kriszta’ line ‘179’. Results are presented as means ± standard deviations. Values of the line ‘179’ were compared to those of the parental wheat genotype Mv9kr1. Gli: gliadin; Glu: glutenin; HMW: high molecular weight glutenins; LMW: low molecular weight glutenins; TOT-AX: total arabinoxylan; UPP: unextractable polymeric protein; WE-AX: water extractable arabinoxylan.

Genotype	Total protein (%)	Total Glu/Gli	UPP (%)	HMW/LMW	TOT-AX (mg/g)	WE-AX (mg/g)
Kriszta	19.00 ± 0.1	1.35 ± 0.0	13.56 ± 1.2	no data	56.45 ± 5.7	24.31 ± 1.0
Mv9kr1	14.90 ± 0.0	0.90 ± 0.0	44.47 ± 0.5	0.77 ± 0.0	42.13 ± 1.4	6.37 ± 0.3
Line ‘179’	17.93 ± 0.6**	0.81 ± 0.0**^††^	39.22 ± 0.3**^††^	1.18 ± 0.0**	59.33 ± 1.0**	10.63 ± 0.2**^††^

**Significantly different from Mv9kr1 at the P < 0.01 level.

^††^Significantly different from ‘Kriszta’ at the P < 0.01 level.

An increase (20.3%) in the total protein content was also measured in the line ‘179’ (Table 3). The glutenin/gliadin ratio decreased, while the ratio of the high molecular weight (HMW) and low molecular weight (LMW) glutenin subunits (HMW/LMW) increased significantly in this genotype compared to Mv9kr1. A significant decrease in the unextractable polymeric protein (UPP) content was also found.

The molecular weights of ω-secalin monomers are very close (48–55 kilodalton)^46^, consequently their separation was only possible by using A-PAGE based on differences in protein charge density at low pH (Supplementary Fig. S3). Bands at identical positions in Mv9kr1 and line ‘179’ proved the Mv9kr1 (maternal) origin of our translocation line. Components of the ω-secalin block encoded by the alleles *Sec-1* (*Gli-R1*) in ‘Kriszta’, ‘Petkus’, ‘Mv Magdaléna’, and line ‘179’ were identical, except that an additional (sixth) faint band was also expressed in the ‘179’.

### Comparative cytogentic analysis of ‘Petkus‘- and ‘Kriszta‘-derived 1RS arms

**Figure 4:**
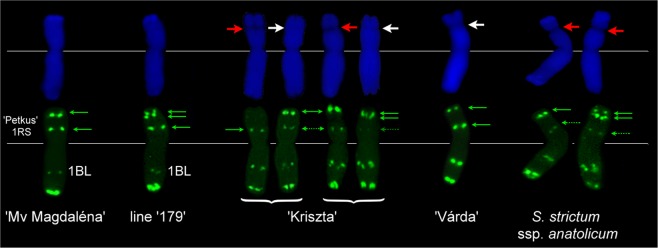
Morphology and FISH hybridization pattern of 1RS chromosome arms of different origin. Upper row (from left to right): DAPI-stained T1BL.1RS translocation chromosome of the wheat cultivar Mv Magdaléna, that of the Mv9kr1-‘Kriszta’ progeny line ‘179’ (as well as ‘C5’ and ‘D5’), 1R chromosomes of rye genotypes ‘Kriszta’, ‘Várda’ and *S. strictum* ssp. *anatolicum*. Differences in the visibility of the secondary constriction (NOR) are indicated with white and red arrows. Lower row: FISH polymorphism with the DNA probe pSc119.2 (indicated with green arrows). Curly brackets indicate chromosome pairs from different plant individuals.

As line ‘179’ showed altered resistance traits compared to ‘Mv Magdaléna’, a study was carried out in order to reveal putative differences between the ‘Petkus’- and ‘Kriszta’-derived 1RS arms at the cytomolecular level. After analysing ten preparations per genotype (‘Mv Magdaléna’, ‘179’, ‘Kriszta’, and its parental genotypes ‘Várda’ and *S. strictum* ssp. *anatolicum* ‘R797’), and ten cells per preparation, we consistently found that 1RS arm of ‘179’ showed a double subterminal pSc119.2 signal on the satellite, while that of ‘Mv Magdaléna’ had a single band at the same position (Fig. 4). Similar double subterminal pSc119.2 pattern was observed in some individuals of the parental *S. cereanum* genotype ‘Kriszta’ that has 1R chromosomes with different pSc119.2 hybridization patterns in homo- or heterozygous form (Supplementary Fig. S4). Among the ‘Kriszta’ plants studied, we detected 1R chromosomes whose FISH pattern was typical for *S. strictum* (‘R797’), but their morphology (size and visibility of the satellite) corresponded to *S. cereale* (‘Várda’), while other 1R chromosomes corresponded morphologically to ‘R797’, but they lacked any hybridization signals at distal position on the satellite (Fig. 4). Based on its pSc119.2 hybridization pattern, we concluded that the 1RS arm of ‘179’ derived from a recombination between that of *S. cereale* (strong interstitial signal proximal to the secondary constriction) and *S. strictum* ssp. *anatolicum* (double sub-terminal pSc119.2 signal).

### SSR marker analysis

The molecular cytogenetic results indicated that the satellite region of the 1RS chromosome arms in ‘179’ and ‘Mv Magdaléna’ are different. In order to estimate the degree of genetic variation between the 1RS arms of ‘179’ and ‘Mv Magdaléna’ in better coverage, we extended the comparative analysis to the whole chromosome arm using 18 PCR-based molecular markers covering the entire length of 1RS (Supplementary Table S3). During the analysis of Mv9kr1, ‘Kriszta’, ‘Mv Magdaléna’ and line ‘179’, 17 out of the 18 markers gave distinct bands in the expected size range (Supplementary Table S4), and no PCR products were amplified from DNA samples of Mv9kr1. Three markers (17.6%) located in the intercalary position (*Xscm9, Xtsm81*)^47^ or on the satellite (*Xtsm120*)^48,49^ generated amplicons polymorphic between ‘Mv Magdaléna’ and line ‘179’ (Supplementary Fig. S5), which indicated that not only the satellite but the interstitial region of the 1RS chromosome arm could be different in the line ‘179’ and ‘Mv Magdaléna’.

### DArTseq analysis and functional annotation

We wanted to investigate allelic composition differences between the 1RS chromosome arms of the T1BL.1RS wheat genotypes ‘179’ and ‘Mv Magdaléna’, as well as between the 1RS arm fixed in the stripe rust resistant lines ‘179’, ‘C5’ and ‘D5’, and the sensitive 1R addition line DA1R in much more detail. For this purpose, a rye DArTseq genotyping platform has been applied which provides much higher marker coverage for 1RS.

After quality filtration by high call rate and reproducibility of the 258,090 Silico-DArT markers obtained in the present study, 175,558 (68%) were scored as ‘0’ (absence of genomic representation) for ‘Mv9kr1’, and as ‘1’ (6401 markers) or ‘0’ (169,157 markers) for the Mv9kr1-*S. cereanum* DA1R line susceptible to stripe rust. Then we selected 5312 putative 1RS-specific Silico-DArT markers using the Mv9kr1-‘Kriszta’ T1BL.1RS genotypes (‘179’, ‘C5’ and ‘D5’) and the DA1R line according to criteria summarized in Supplementary Data S1. We obtained a total of 71,177 SNP markers, as well. After quality filtration and removing markers having alleles in the wheat genotype ‘Mv9kr1’, 6027 markers remained of which 1755 were selected as putative 1RS-specific SNP markers.

**Figure 5:**
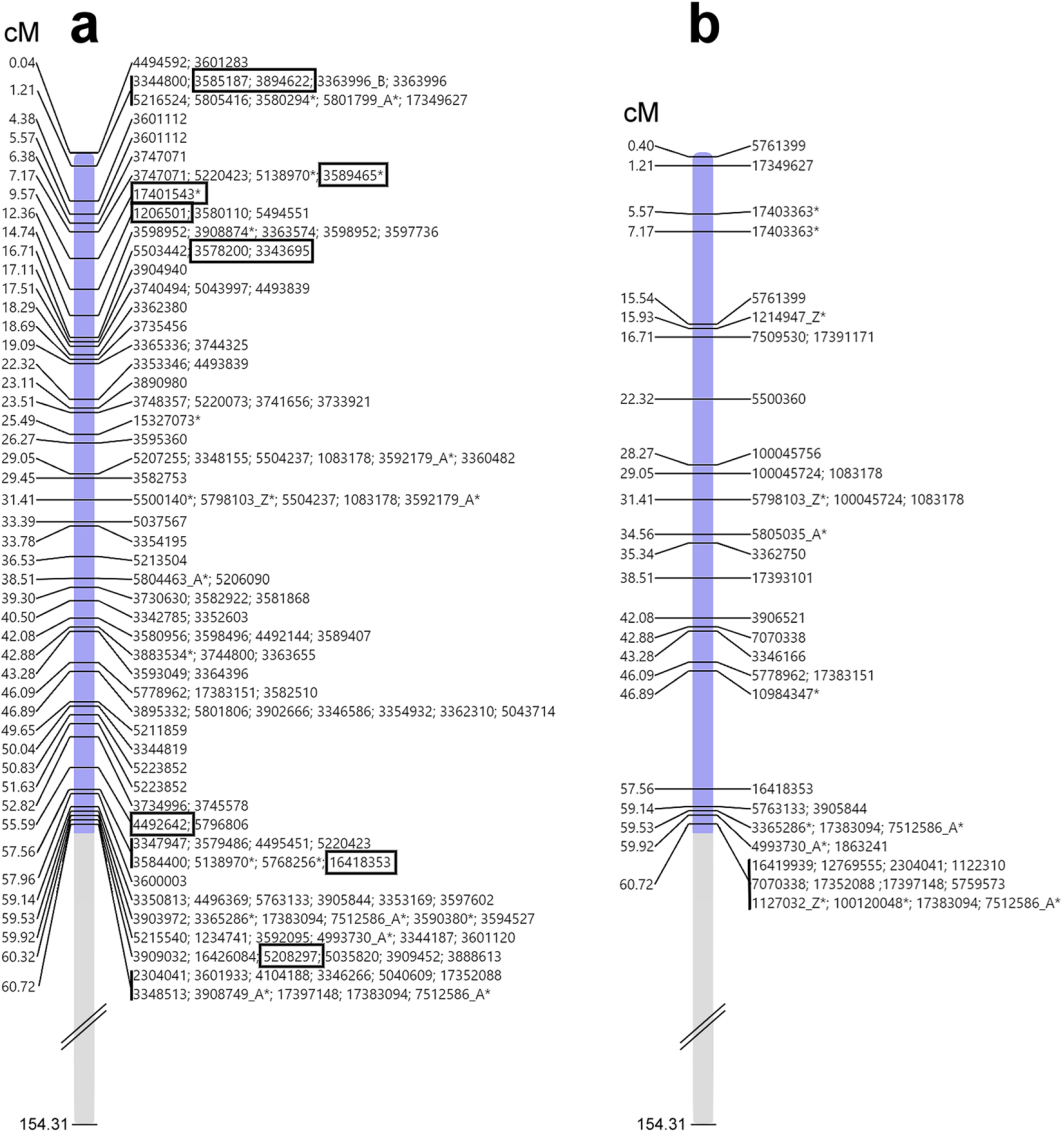
Genetic linkage maps for the rye chromosome arm 1RS of line ‘179’. (**a**) Distribution of the Silico- and SNP-DArT markers specific for the stripe rust resistant ‘179’. Markers linked to LRR domain are enclosed by black rectangles. (**b**) Distribution of 1RS-specific markers exhibiting allelic variation between ‘Kriszta’ and ‘Petkus’ (present in ‘Mv Magdaléna’) 1RS. 1RS and 1RL (lacking from the T1BL.1RS translocation) chromosome arms are indicated in blue and gray, respectively. The marker distance is expressed in centimorgans (cM) from the telomere of 1RS (0 cM) to the putative centromere (60.72 cM) of chromosome 1R. *SNP-DArT markers.

In order to confirm that the selected Silico- and SNP-DArT markers are specific for rye chromosome 1R, we used trimmed marker sequences for BLASTn against the rye Lo7 genomic sequences. Out of the 5312 Silico-DArT markers, 679 were aligned to the rye Lo7 WGS 1R contigs, while out of the 1755 SNP markers 168 gave hits against 1R contigs of rye (Supplementary Data S2). These (679 + 168 = ) 847 markers were used in the subsequent analysis to select markers specific for the short arm of 1R, and to order them using the recent version of the rye virtual gene order map (Rye Genome Zipper v.2)^50^. As Bauer *et al*. (2017)^50^ mapped the 1R centromere to the position of 60.72 cMorgan (cM), we checked the region ranging from 0 cM to 60.72 cM (considered as 1RS) for the presence of marker-specific Lo7 WGS contigs. Out of the 847 1R-specific markers mentioned above, 233 (27.5%) were located on the short arm of 1R. Out of the 233 markers, 129 (111 Silico-DArT + 18 SNP; 55.36%) were specific for the stripe rust resistant genotypes ‘179’, ‘D5’, and ‘C5’ (Fig. 5a), and 104 markers (44.64%) were specific for the susceptible DA1R line. This pronounced difference in the allelic composition suggests that the 1RS arm fixed in the stripe rust resistant genotypes is different from that of DA1R.

We also compared the allelic composition of the 1RS arm of the line ‘179’ with the ‘Petkus’-derived 1RS of ‘Mv Magdaléna’. Out of the above-mentioned 233 1RS-specific markers 225 gave unambiguous allelic results in both wheat genotypes. Thirthy-seven (16.4%) of the 225 markers showed different alleles in ‘179’ relative to ‘Mv Magdaléna’ (Fig. 5b).

Theoretically, a significant amount of the DArTseq markers is associated with coding sequences or other parts of the “genic” region (promoters, introns, 3′ UTR sequences), which is the consequence of a methyl filtration step (complexity reduction) during the process of DArTseq marker production. After homology search of the 233 1RS-specific markers against the cDNA sequences of rye or the related cereal species, we obtained significant hits for 162 (135 Silico-DArT and 27 SNP) (69.5%) markers (Supplementary Data S2). Out of the 162 marker-specific genes, 108 and 142 showed hits to the HMM based Pfam database at the level of superfamilies and families, respectively, and 112 were functionally annotated to Gene Ontology (GO) terms (Supplementary Data S2).

**Figure 6:**
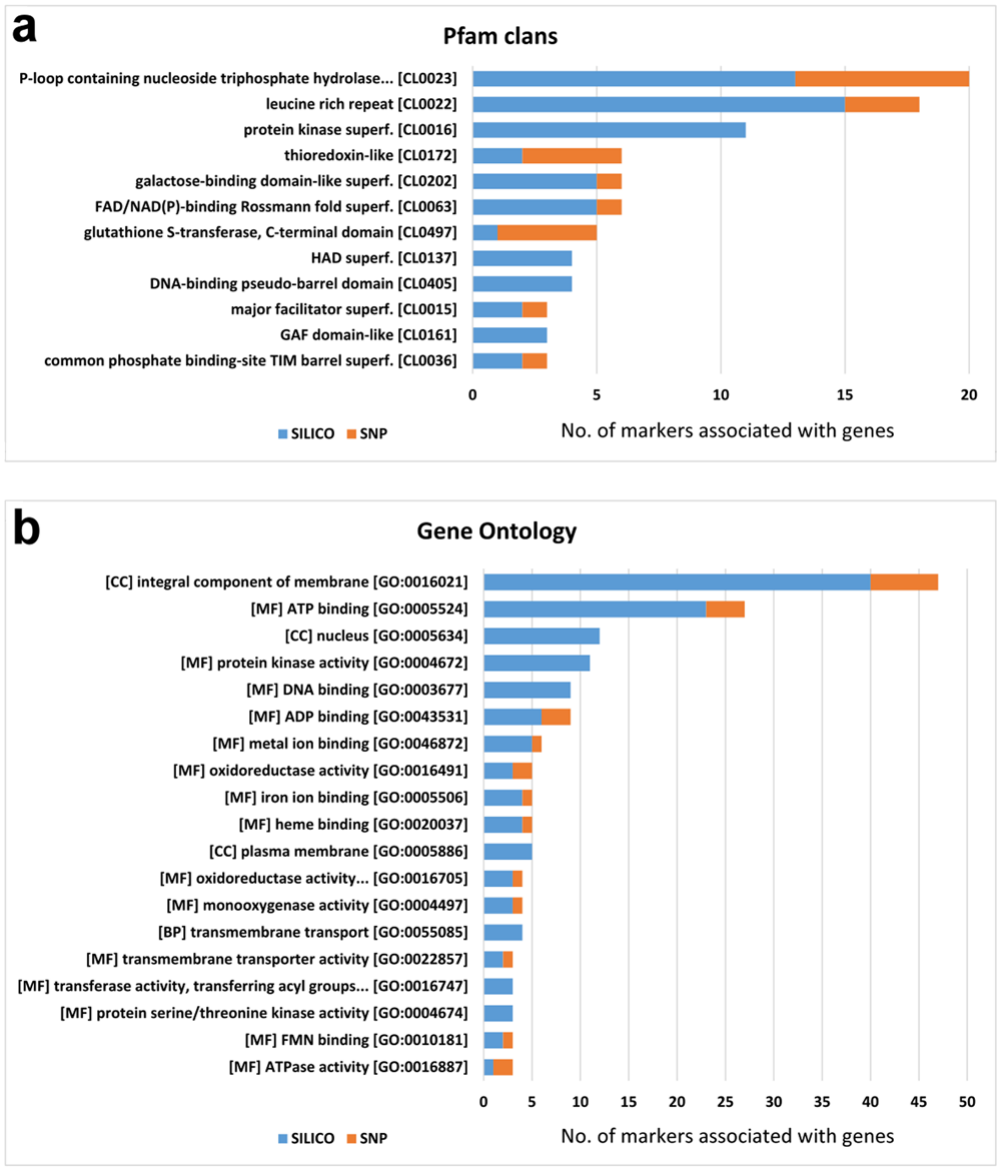
Functional annotation of 1RS-specific and gene-associated DArTseq markers. (**a**) Pfam high-level grouping- and (**b**) Gene Ontology-based functional annotation of 203 Silico- and 30 SNP-DArT markers. Figures present groups with at least 3 members. The complete datasets are available in the Supplementary Data S2.

The protein domain analysis revealed that the ‘P-loop containing nucleoside triphosphate’ [P-loop_NTPase; CL0023] and the ‘Leucine Rich Repeat’ [LRR; CL0022] were the most common and most over-represented superfamilies. Moreover, the GO analysis suggested that many of these proteins are also associated with the ‘integral components of the membrane’ [GO:0016021], ‘ATP binding’ [GO:0005524], ‘ADP binding’ [GO:0043531] and ‘protein kinase activity’ [GO: 0004672] terms. These domains and gene ontologies were found to be typical of most disease resistance proteins in rye. The most represented superfamilies and functional groups with at least 3 members are visualized in Fig. 6.

We found 10 markers polymorphic between the susceptible (DA1R) and stripe rust resistant (‘179’, ‘C5’ and ‘D5’) genotypes, and associated with genes coding LRR domains (Fig. 5a). These markers located on the subtelomeric (3585187, 3894622, 3589465, 17401543, 1206501, 3578200 and 3343695) or the pericentromeric (4492642, 16418353 and 5208297) regions homologous to the 1.21–16.71 cM and 55.59–60.32 cM intervals, respectively, of the 1RS chromosome arm.

## Discussion

Line ‘179’ presented in this study provides new allelic variations for the rye chromosome arm 1RS introgressed into wheat as it carries a pair of T1BL.1RS chromosomes in which the 1RS arm derives from the perennial rye cultivar ‘Kriszta’, a hybrid of *S. cereale* and *S. strictum* ssp*. anatolicum*.

### Effects on morphological and quality traits

The novel genetic variation on the 1RS arm of line ‘179’ caused positive changes in tillering capacity, lentgth of spikes, number of spikelets, and fertility that led to a 72–87% increase in the number of seeds per plant, confirming the findings of previous studies^34^.

Replacement of 1BS with 1RS in wheat leads to quality deficiencies. Changes in the protein composition, particularly the reduction in the concentration of low-molecular-weight (LMW) glutenin and gliadin proteins, cause weaker dough strength and stickiness^51^. Although Kumlay *et al*. (2003)^52^ found that the presence of the secalin genes had greater negative impact than the loss of *Glu-B3* and *Gli-B1* loci on 1BS, contribution to the effects mentioned above of *Sec-1* alleles is not entirely clear^53^. Analysing highly informative quality parameters in doubled haploid T1BL.1RS translocation lines it was concluded that the lower quality caused by the translocation chromosome can be counterbalanced by beneficial alleles at *Glu-A1*, *Glu-B1* and *Glu-D1*, particularly in lines with high protein content^54^. In this respect, line ‘179’ may be a promising breeding material having elevated total protein content and a unique A-PAGE profile of ω-secalins. Amino acid sequence analysis revealed that ω-secalin genes from rye (*S. cereale*), hexa- and octoploid triticale, and T1BL.1RS translocation lines are homologous and highly conserved^55^. Appearance of a new gene product in line ‘179’ indicates that alleles at the *Sec-1* locus are different from those in *S. cereale*, and makes it worth studying the bread-making quality of this genetic material in the future. Moreover, the presumably decreased bread making quality of ‘179’ might be improved by the transfer of its T1BL.1RS translocation into an advanced wheat variety with good rheological and baking properties.

Line ‘179’ is rich in arabinoxylans, a major component of dietary fibers (DF) in wheat and rye. Testing several sets of wheat-rye addition and translocation lines, Boros *et al*. (2002)^56^ found that rye chromosomes 2R, 5R and 6R were associated with significantly increased amounts of soluble AX, while the additions of 1R, 3R, 4R and 7R resulted in significantly lower levels of soluble DF and AX. Contrary to this, Cyran *et al*. (1996)^57^ reported that chromosomes 4R had an impact on high AX expression in wheat comparable to that of rye AX content. Our results suggest that the role of chromosome arm 1RS in increasing both TOT-AX and WE-AX content in wheat is also significant, presumably due to the presence of *S. strictum* chromatin

On the basis of our findings we can conclude that the line ‘179’ carrying a new T1BL.1RS translocation is an important genetic resource for breeding programmes aimed at producing wheat cultivars with improved grain yield and increased level of dietary fibers.

### Effects on disease resistance

Stripe rust, earlier considered infectious in regions of lower temperature, nowadays causes severe epidemics in warmer wheat growing areas (including Central Europe) as well, owing to the emergence of new races with expanded virulence profiles and increased aggressiveness especially at higher temperature^58^. Identification and incorporation of diverse sources of resistance (particularly of the durable type) against this pathogen is an urgent and challenging task for resistance breeding. So far several successful attempts exploiting the genetic variation of wild and cultivated relatives have been reported to improve disease resistance of bread wheat (for review see^1^). Regarding rye genepool as resistance source for wheat improvement, Wang *et al*. (2009)^59^ transferred 1RS arm from the rye cultivar German White into the chinese wheat cultivar Xiaoyan 6, and produced a T2BL.1RS wheat-rye chromosome translocation line resistant to stripe rust and powdery mildew. Using Chinese wheat and rye cultivars, various T1BL.1RS translocation lines resistant to stripe rust^60^ or both stripe rust and powdery mildew^61–63^ were also developed. In case of wild relatives of rye, only two papers reporting on the transfer of stripe rust resistance from *S. africanum* have been published^64,65^, where the resistance genes were mapped on the chromosome arms 1RS^64^ and 2RL^65^.

In the present study, the novel T1BL.1RS translocation line ‘179’ was resistant not only to stripe rust, but also to leaf rust, which is consistent with the fact that resistance genes *Lr26* and *Yr9* (together with *Sr31*), form a closely linked gene cluster mapped on the satellite of 1RS arm in *S. cereale*^12,66^. It is well known that LRR domains are important parts of the plant immune receptors^67^. Consistently with this, we found that DArTseq markers associated with genes carrying the LRR domain, and polymorphic between the stripe rust resistant ‘179’ and the sensitive DA1R were located on the subtelomeric 1.21–16.71 cM interval of Lo7 1RS arm. For the time being we are uncertain whether the genes providing resistance against stripe rust and leaf rust in the genotype ‘179’ are variants of *Yr9* and *Lr26* or they are new resistance genes. Genetic mapping of resistance against stripe rust and leaf rust using an M9kr1-‘Kriszta’ T1BL.1RS line ‘179’ (resistant) x M9kr1-‘Kriszta’ DA1R (susceptible) biparental mapping population will help to identify the position of the resistance genes in the line ‘179’. Later, production of knockout mutants in ‘179’, sequencing of T1BL.1RS chromosomes flow-sorted from the resistant genotype ‘179’ and from its susceptible mutants (MutChromSeq), and combination of the sequences with the mapping data will open the way for the positional cloning of the resistance genes introgressed into the line ‘179’^68^.

The explanation why the M9kr1-‘Kriszta’ T1BL.1RS line ‘179’ is resistant to stripe rust and leaf rust while the M9kr1-‘Kriszta’ DA1R line is susceptible may be that diverese 1R chromosomes exist in the open-pollinated population of parental rye cultivar Kriszta, and the 1RS arm fixed in the stripe rust resistant genotypes is different from that fixed in the Mv9kr1-‘Kriszta’ DA1R line. This idea was supported by the fact that more than half (55.36%) of the 233 1RS loci identified by the DArTseq technology were specific for the resistant genotype, and 44.64% were specific for the susceptible 1R addition line. This supposition was further supported by the cytomolecular results that different 1R chromosomes with altered pSc119.2 FISH patterns were detected in ‘Kriszta’ rye, which agreed well with a previous study, where four out of five’Kriszta’ plants investigated by the 1RS-specific SSR marker *RMS13* showed polymorphic amplicon patterns^16^. Based on this finding, it is assumable that during meiosis in the descendants of the *S. cereale* × *S. strictum* ssp. *anatolicum* hybrid several crossing-over events occurred resulting in different 1RS chromosome arms that carry chromatins from the two species in various proportions. Our hypothesis on the recombination was further confirmed by molecular marker analyses. Due to the high transferability of SSR markers between *S. cereale* and *S. strictum*^45^ these markers were suitable to compare 1RS arms originated from *S. cereanum* and *S. cereale*. Three of the 17 SSR markers (17.6%) unambigously detected polymorphic loci on the 1RS arm of line ‘179’ relative to the ‘Petkus’ type 1RS in ‘Mv Magdaléna’. Interestingly, we obtained a similar level of polymorphism (16.4%) between the 1RS loci in ‘179’ and ‘Mv Magdaléna’ when DArTseq markers were used in higher coverage for genotyping (37 out of the 225 were polymorphic).

Knowing that the *Pm8* locus (in *S. cereale*) is mapped only 1.7 cM distal to the *Lr26/Sr31/Yr9* gene cluster^69^, and the artificially inoculated ‘Kriszta’ was resistant to powdery mildew, the susceptibility of line ‘179’ was unexpected. The most probable explanation of this observation may be the phenomenon of genetic suppression of resistance genes, which often manifests itself when these genes are transferred from related species with lower ploidy level to hexaploid bread wheat^70–72^ or synthetic wheat^73,74^. Expression of the 1RS-derived *Pm8* is suppressed by translated gene products from the *Pm3* locus, a wheat ortholog of *Pm8*, located on wheat chromosome arm 1AS^75^. Resistance suppression or non-suppression of *Pm8* depends on the wheat germplasm in which the 1RS chromosome arm is introgressed^13,76,77^. Based on the findings of the reports mentioned above, we have speculated about the following possibilities: (i) suppression activity of *Pm3* on chromosome arm 1AS in wheat genotype Mv9kr1 (in contrast with many Chinese wheat cultivars) is expressed, (ii) the gene on 1RS in line ‘179’ must be *Pm8* carrying allelic variation for resistance against powdery mildew, (iii) if line ‘179’ carries a gene of *S. stricum* origin different from *Pm8*, its expression is also influenced by the gene products of *Pm3*.

### Final conclusion

The *S. cereale* x *S. strictum* recombinant 1RS originating from *Secale cereanum* cv. Kriszta confers novel genetic diversity in the T1BL.1RS translocation chromosome of line ‘179’ reported here. The new, morphologically uniform wheat genotype possessing altered spike morphology, increased fertility, high protein and arabynoxylan content, and resistance to stripe rust and leaf rust is a promising gene source for wheat breeding. It has been involved in a breeding program aiming transfer of the new T1BL.1RS translocation into modern winter wheat cultivars, therby it can contribute to healthy eating and a more cost-efficient wheat production. Line ‘179’ will also facilitate the map-based cloning of the resistance genes located on the recombinant 1RS chromosome arm.

## Methods

For reasons of clarity, a short overview of the aims as well as methods and genotypes used in the present study is given in Table 1.

### Plant materials

The following plant materials were used in the present study: *Secale cereanum* (2n = 2x (x - eks) = 14; RR) cv. Kriszta; accession R797 of *S. strictum* (C. Presl) C. Presl. ssp. *anatolicum* (Boiss.) K. Hammer (2n = 2x (x - eks) = 14; RR) from the Genebank of IPK Gatersleben; *S. cereale* (2n = 2x (x - eks) = 14; RR) cv. Várda; winter wheat (*Triticum aestivum* L. 2n = 6x (x - eks) = 42; AABBDD) line Martonvásári 9 kr1 (Martonvásári 9/Chinese Spring//Martonvásári 9; Mv9kr1) carrying recessive crossability allele *kr1* from ‘Chinese Spring’^78,79^; spring wheat genotype ‘Chinese Spring’; Martonvásár T1BL.1RS wheat cultivar Mv Magdaléna (Yubileinaya 50/Fundulea 29//MVMA; T1BL.1RS of Fundulea 29 derives from ‘Petkus’ rye^16^); wheat-*S. cereanum* 1R disomic addition line (Mv9kr1/Kriszta//Mv9kr1; DA1R) identified earlier^45^, and wheat-*S. cereanum* T1BL.1RS translocation lines ‘179’, ‘C5’ and ‘D5’ (Mv9kr1/Kriszta//Mv9kr1) developed in the present study.

### Development of wheat-S. cereanum T1BL.1RS translocations

Flow diagram of the development process is presented in Supplementary Fig. S1. The wheat-*S. cereanum* T1BL.1RS translocations cytogenetically identified in the BC_2_F_8_ generation have been multiplied in the field since then.

### Preparation of metaphase chromosome spreads

Chromosome preparations from roots of germinating seeds of ‘Várda’,’R797’, ‘Kriszta’, lines ‘179’, ‘C5’, ‘D5’, and’Mv Magdaléna’ followed the method described by Endo and Gill (1984)^80^ with minor modifications. Roots kept in Carnoy’s solution I (absolute ethanol:glacial acetic acid 3:1 v/v) at 37 °C for seven days were stained with 1% (w/v) acetocarmine for at least 2 h, fixed again and stored at −20 °C until use. Root tips were squashed in 45% acetic acid. After removing the coverslips by freezing in liquid nitrogen, the preparations were dehydrated in ethanol series, air-dried overnight and stored at −20 °C.

### Fluorescence and genomic *in situ* hybridization

Total rye (*Secale cereale* L.) genomic DNA was labeled with digoxigenin-11-dUTP (Roche Diagnostics, Mannheim, Germany) by nick translation and used as a probe for genomic *in situ* hybridization (GISH). Rye and wheat chromosomes were identified with the rye subtelomeric heterochromatic sequence pSc119.2^31^ and FISH probe combination of pSc119.2, Afa-family^81^ and the 45 S rDNA clone pTa71^82^, respectively. PCR-amplified pSc119.2^83^ and Afa-family were labeled with biotin-16-dUTP (Roche) and digoxigenin-11-dUTP, respectively, while pTa71 was co-labeled with these nucleotides. GISH and fluorescence *in situ* hybridization (FISH) were carried out simultaneously. The hybridization mixture per slide (total volume = 30 µL) contained 30 ng labeled rye genomic DNA, 50 ng pSc119.2 repetitive DNA probe, 50% v/v formamide, 2 × SSC (0.15 mol/L NaCl plus 0.015 mol/L sodium citrate), 10% w/v dextran sulphate, 1.4 µg salmon sperm DNA and 0.1% w/v sodium dodecyl sulphate. The mixture did not contain wheat blocking DNA. Chromosome preparations were denatured at 75 °C for 6 min and hybridized overnight at 42 °C. Signals of the digoxigenin- and biotin-labeled probes were detected using anti-digoxigenin-Rhodamin Fab fragments (Roche) and streptavidin-FITC (Roche), respectively. Slides were counterstained with 1 µg/mL DAPI (4’,6-diamidino-2-phenylindole, Amersham, Germany). Images were acquired through a Zeiss Axioskop 2 fluorescence microscope equipped with filter sets appropriate for DAPI (filter set 1), FITC (filter set 10), Rhodamin (filter set 15) and for the simultaneous detection of FITC and Rhodamine (double filter set 24) with a Spot CCD camera (Diagnostic Instruments, Sterling Heights, MI, USA) and processed with Image-Pro Plus software (Media Cybernetics, Silver Spring, Md, USA).

Observations were carried out on ten preparations (i.e. ten individuals) of each genotype and on ten cells per preparation.

### Field observations

As stripe rust isolates for artificial inoculation are not available in Hungary, assessment and comparision of infection responses of the lines ‘179’, ‘C5’ and ‘D5’, the T1BL.1RS cultivar Mv Magdaléna, the disomic addition line DA1R carrying a whole 1R chromosome as well as the parental genotype ‘Kriszta’ was only possible under field conditions. In order to ensure the natural infection, the genotypes mentioned above and the highly susceptible parental Mv9kr1 (as possible infection source) were grown in neighbouring plots (2 m^2^ each) of the high-input pre-breeding (PB) nursery of the Agricultural Institute, Centre for Agricultural Research (Martonvásár, Hungary; geographic coordinates: 47°19'58“N 18°47'08“E) in three consecutive growing seasons from 2014 to 2016. Infection severity (0–100%) was scored according to the modified Cobb scale^84^ based on the percentage of infected leaf area. Ratings were recorded weekly for a month from the first appearance of the disease on the susceptible Mv9kr1.

Morphological comparision of line ‘179’ with the parental wheat genotype Mv9kr1 was carried out in the PB nursery in 2015, and in both the PB nursery and a low-input location (LI; Tükrös nursery, Martonvásár, Hungary; 47°18'40“N 18°46'56“E) in 2019. Fifty seeds of each genotype were sown in 1 m rows with 10 seeds per row and a row distance of 15 cm. Characteristics were taken from ten randomly selected plants of each genotype. Plant height and tillering (spikes per plant) were measured in the field, the length of the main spike, number of spikelets per main spike, number of seeds per main spike, number of seeds per plant and thousand kernel weight (TKW) were recorded after harvest.

### Quality measurements

Crude protein content was assessed from whole grain flour of 40 mg seeds per genotype by the Kjeldahl method^85^ using Kjeltec 1035 Analyzer. Glutenin, gliadin, albumin + globulin, and unextractable polymeric protein (UPP% = insoluble glutenin/soluble + insoluble glutenin) content were determined by size-exclusion high-performance liquid chromatography (SE-HPLC)^86^, relative amounts of the HMW glutenin subunits were determined by reversed-phase high-performance liquid chromatography (RP-HPLC)^87^, while total and water extractable pentosans (arabinoxylans - AX) were determined by colorimetry using modified methods described earlier in details^88^.

### Electrophoresis of prolamin storage proteins

Prolamin storage protein (gliadin and/or secalin) composition of wheat genotypes Chinese Spring (as reference), Mv9kr1, ‘Mv Magdaléna’ and line ‘179’, and rye cultivars ‘Kriszta’ and ‘Petkus’ was determined by acid-polyacrylamid gel electrophoresis (A-PAGE, pH = 3.1) using the method of Jackson *et al*. (1996)^89^. Prolamins were extracted from single seeds with 70% ethanol at 65 °C shaken for 30 minutes. Samples were run on 12,5% polyacrylamide gel with an acrylamide/bisacrylamide ratio of 1:32. Gel polymerization was initiated by hydrogen peroxide. Gels were run on 30 mA for 10 minutes than on 70 mA for 3 hours using reverse poles. Gels were dyed with Brilliant Blue G solution (Sigma).

### Artificial disease resistance tests

Resistance to leaf rust (*Puccinia triticina*) and powdery mildew (*Blumeria graminis* f.sp. *tritici*) infection of the introgression line was tested under greenhouse conditions on 10-day-old seedlings of ‘Mv Magdaléna’, line ‘179’, the susceptible Mvkr1 and the resistant ‘Kriszta’. Pathotype populations with known virulence spectrum are presented in Supplementary Table S2 online. Before brush-inoculation with water-based leaf rust urediospore suspension, the wax layer was washed off from the leaf surface. To ensure high humidity conditions, the inoculated plants were covered with polythene bags for 48 hours. Infection types were recorded 14 days after inoculation using the 0 to 4 Stakman-scale^90^.

In the case of powdery mildew, the isolates (LH07–14, LH14–14) were formerly propagated on a susceptible wheat cultivar (Carsten V). Ten plants of each genotype were grown in a 50 × 40 cm wooden isolator box with 80–90% relative air humidity, and 16 h daily natural and illuminated light conditions at 18 °C. Heavily sporulating colonies on ‘Carsten V’ plants were shaken into the test box. Infection levels were scored on a 0 to 4 scale (0–2: resistant, 3–4: susceptible)^91^ after ten days.

### SSR and ISBP marker analyses

Genomic DNA was extracted from fresh young leaves at the 4-leaf stage from ‘Kriszta’, Mv9kr1, ‘Mv Magdaléna’ and line ‘179’ using QuickGene Mini80 extraction system and QuickGene DNA tissue kit S (FujiFilm, Japan). The thirteen SSR (Simple Sequence Repeat) and five ISBP (Insertion Site Based Polymorphism) markers used in this study are listed in Supplementary Table S4 online. PCR reactions were carried out in a volume of 15 µL in a Mastercycler Nexus Gradient Thermal Cycler (Eppendorf, Hamburg, Germany). The reaction mixture contained 45 ng of template DNA, 5 µL of 10x PerfectTaq Plus PCR Buffer (5 PRIME, Hilden, Germany), 0.2 mmol/L of each dNTP, 0.1 µmol/L each of the forward and reverse primers, and 0.375 U of PerfectTaq Plus DNA Polymerase (5 PRIME, Hilden, Germany). Touchdown PCR was performed using the following reaction profiles: 94 °C (3 min), 6 cycles of [94 °C (20 sec), 65 °C (20 sec), 72 °C (35 sec)], 35 cycles of [94 °C (15 sec), primer annealing temperature (20 sec), 72 °C (35 sec)], 72 °C (2 min), hold at 15 °C. PCR amplicons were separated using a Fragment Analyzer Automated CE System equipped with a 12-Capillary Array Cartridge (effective length 33 cm) (Advanced Analytical Technologies, Ames, IA, USA). Fragment length differences were measured and documented using PROsize v2.0 software.

### DArTseq marker analysis

Genomic DNAs were extracted from line DA1R, Mv9kr1-‘Kriszta’ T1BL.1RS lines ‘179’, ‘C5’, and ‘D5’, as well as wheat varieties Mv9kr1 and ‘Mv Magdaléna’ the same way as described for the SSR and ISBP marker analysis. DNA samples (30 μl at a concentration of 70 ng/μl each) were sent to Diversity Arrays Technology Pty, Ltd. (University of Canberra, Australia) and genotyped with Rye DArTseq version 1.0. The generated results were listed in tables for Silico- and SNP-DArT markers. Markers with call rate > 95% [available from the BioStudies database (https://www.ebi.ac.uk/biostudies/studies/S-BSST280; Accession number: S-BSST280)] were selected for subsequent analysis. Silico-DArT markers were scored as binary data (1 or 0) reflecting the presence or absence of a marker in the genomic representation of each sample^92^. We used 1-row Mapping Format for the SNP markers scored as ‘−’, ‘0’, ‘1’ and ‘2’ representing absence of a marker, reference allele (Rye_v2; provided by Diversity Arrays Technology Pty Ltd.) only, alternative allele, and both reference and alternative alleles, respectively. Our objective was to identify markers in order to differentiate between wheat chromosomes and chromosome 1R of *S. cereanum*, and investigate polymorphism between stripe rust resistant and stripe rust susceptible wheat-*S. cereanum* lines. In order to separate markers specific for *S. cereanum*, Silico-DArT markers, as well as SNP alleles present in the wheat (Mv9kr1) genome were discarded. Genotyping data of the Mv9kr1-*S. cereanum* T1BL.1RS (‘179’, ‘C5’, ‘D5’) and 1R addition (DA1R) lines were then analysed to select putative 1 R specific markers, as well as markers polymorphic between stripe rust resistant (‘179’, ‘C5’, ‘D5’) and stripe rust susceptible (DA1R) genotypes using criteria summarized in Supplementary Data S1 online.

Genomic and cDNA sequence sets of *S. cereale* (IPK Gatersleben, https://webblast.ipk-gatersleben.de/ryeselect/downloads; Lo7_WGS_contigs_v2 and MIPS_CDS_mar14, respectively)^50^ were retrieved and used to identify the chromosomal location and gene associations of the putative 1R markers. Trimmed sequences of the putative 1R markers were used as querries for sequence similarity search performed using the *blastn* package of the BLAST Command Line Application 2.9.0 (ftp://ftp.ncbi.nlm.nih.gov/) with the following parameters: E-value = 1e^−5^; -max_target_seqs = 1; -max_hsps = 1.

Translated cDNA sequences were used for functional annotations. The Gene Ontology information was extracted from the Universal Protein Resource (ftp://ftp.uniprot.org; UniProt release 2019_04) database, the resulting protein collections were subsequently scanned with the Hidden Markov Model (HMM)-based HMMER 3.0 software package (http://eddylab.org/software/hmmer/)^93^ on the Pfam 32.0 entries (ftp://ftp.ebi.ac.uk). Results of the homology search and functional annotations were summarized in Supplementary Data S2 online.

In order to obtain a linear order of DArTseq markers for the chromosome 1R, the best hits against Lo7 WGS contigs v2 were used to incorporate 1R-specific DArTseq markers into Rye Genome Zipper (Rye Genome Zipper v2.; DOI:10.5447/IPK/2016/58)^50^. CentiMorgan (cM) values of the marker-specific 1R contigs obtained from the rye GenomeZipper were used to order the markers. Their positions on the 1R genetic map were visualized using a custom-made software (Supplementary Data S3).

### Statistical analysis

Significant differences between line ‘179’ and the wheat parent Mv9kr1 were calculated from 10 measurements in the case of morphological characteristics, and from 3 replications in the case of compositional properties by means of one-way ANOVA function of the IBM SPSS Statistics 20.0 software (SPSS Inc., Chicago, IL, USA).

## Supplementary information

Supplementary information.

Supplementary information2.

Supplementary information3.

Supplementary information4.

## Data Availability

The datasets analysed during the current study are available in the BioStudies repository [https://www.ebi.ac.uk/biostudies/studies/S-BSST280] with the accession code S-BSST280, and also included in supplementary information files (Supplementary Data S1–S3) of this article.
