# Supplementary material for: 1RS arm of Secale cereanum ‘Kriszta’ confers resistance to stripe rust, improved yield components and high arabinoxylan content in wheat
Source: Sci Rep. 2020 Feb 4;10:1792. doi: 10.1038/s41598-020-58419-3 (PMC7000720; doi:10.1038/s41598-020-58419-3)
Supplement: Supplementary file 1 — Supplementary information. [file 41598_2020_58419_MOESM1_ESM.docx]

**Supplementary Information (Supplementary Reference List)**

**1RS arm of *Secale cereanum* ‘Kriszta’ confers resistance to stripe rust, improved grain yield and high arabinoxylan content in wheat**

Éva Szakács^1,#^, Kitti Szőke-Pázsi^1,#^, Balázs Kalapos^1^, Annamária Schneider^2^, László Ivanizs^1^, Marianna Rakszegi^1^, Gyula Vida^1^, István Molnár^1,3,*^ and Márta Molnár-Láng^1^

^1^Agricultural Institute, Centre for Agricultural Research, Martonvásár, H-2462, Hungary

^2^ National Food Chain Safety Office, Budapest, 1024 Hungary

^3^Institute of Experimental Botany, Center of the Region Haná for Biotechnological and Agricultural Research, Šlechtitelů 31, CZ-78371 Olomouc, Czech Republic

*corresponding author e-mail: molnar.istvan@agrar.mta.hu

^#^these authors contributed equally to this work

**Supplementary Figure S1** Flow diagram of the development of Mv9kr1-‘Kriszta’ T1BL.1RS translocation lines.

**
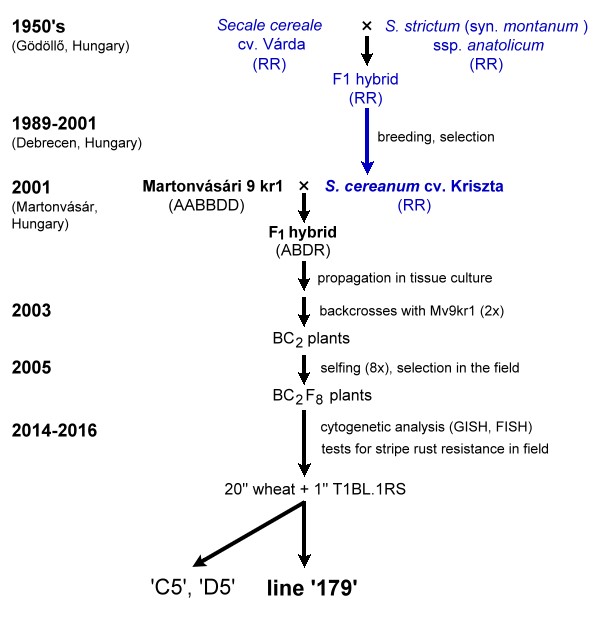
**

**Supplementary Figure S2** Spike and seed morphology. (**A**) Parental wheat line Mv9kr1, (**B**) parental rye (*S. cereanum*) cultivar Kriszta, (**C**) Mv9kr1-‘Kriszta’ (wheat-*S. cereanum*) line ‘179’. Images of seeds are resized proportionally.

**
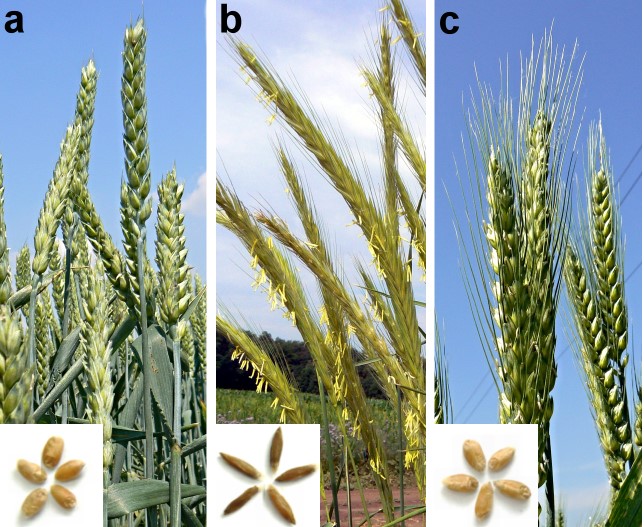
**

**Supplementary Figure S3** A-PAGE electrophoregram of wheat and rye gliadins. Lane 1: parental wheat genotype Mv9kr1; lanes 2 to 4: line ‘179’; lanes 5 to 7: ‘MvMagdaléna’ carrying 1RS from ‘Petkus’; lanes 8 to 10: rye cv. Kriszta; lanes 11 to 13: rye cv. Petkus; lane 14: wheat cv. Chinese Spring (as a reference genotype). Arrowheads indicate 1RS-encoded ω-secalin (*Sec-1*) bands **(Kozub et al. 2018)** vertical arrows point to an additional band appeared in line ‘179’. Two gliadin bands characteristic to Mv9kr1 are indicated by asterisks. Wheat-specific gliadins band under *Gli-A1x* are not presented.

**
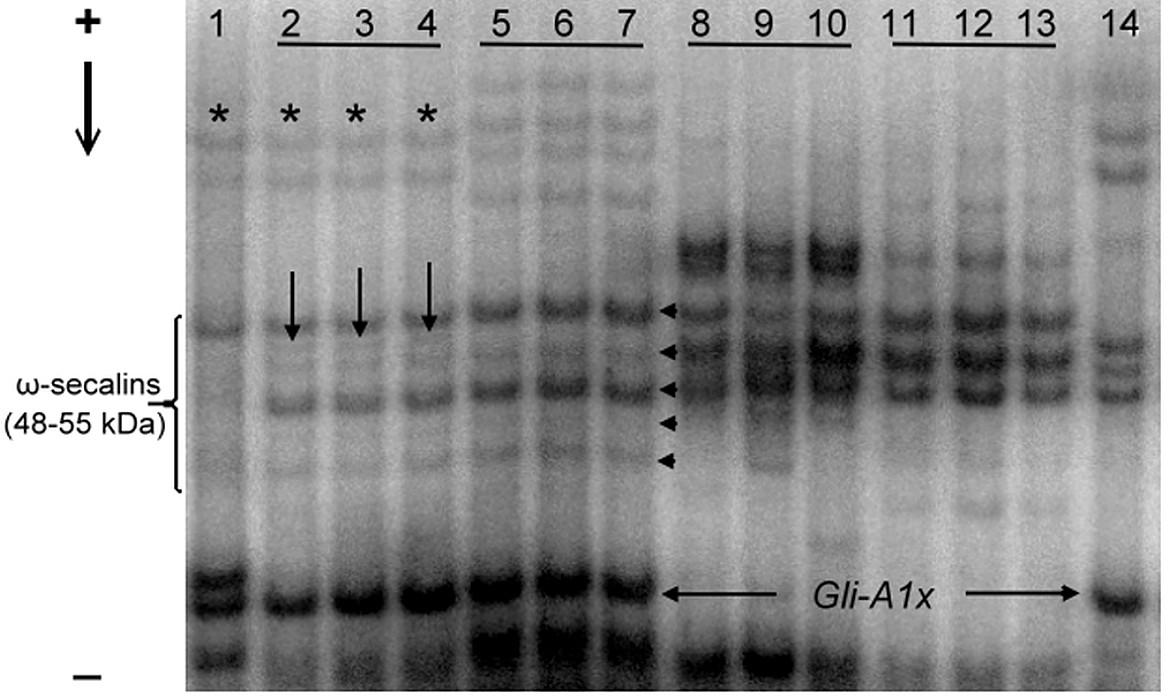
**

**Supplementary Figure S4** FISH pattern of the rye cultivar Kriszta. Chromosome pairs having polymorphic hybridization sites (1R, 4R, 7R) with the repetitive DNA probe pSc119.2 (green) are indicated with asterisks. Heteromorphic 1R chromosomes (arrowed) are enlarged at the top-right corner. The mitotic chromosome spread was counter stained with DAPI (blue). Scale bar: 10µm


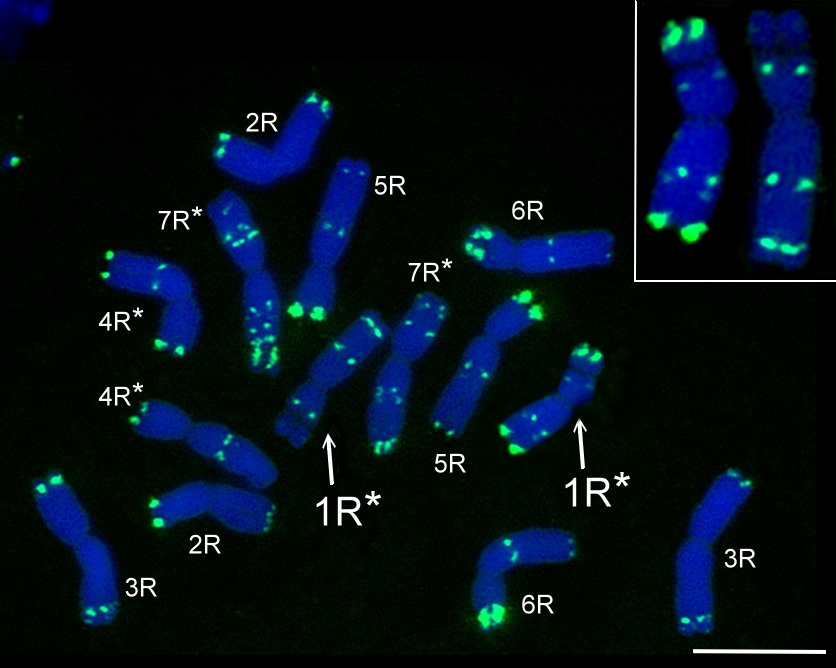


**Supplementary Figure S5** Results of the molecular marker analysis. (**a**) 1RS chromosome-specific PCR amplification patterns of SSR markers *Xscm9*, *Xtsm81* and *Xtsm120* from DNA templates of the parental wheat line Mv9kr1 (Mv9), parental perennial rye ‘Kriszta’ (Kr), wheat cultivar Mv Magdaléna (Mag) carrying T1BL.1RS translocation of ‘Petkus’ rye origin, and Mv9kr1-‘Kriszta’ introgression line ‘179’ (179). PCR products showing polymorphism between ‘Mv Magdaléna’ and line ‘179’ are marked with arrows. (**b**) Monomorphic bands of ISBP markers *Xora6*, *Xora7* and *Xora11* on the same DNA samples. Images (**a**) and (**b**) are separated by two horizontal lines. (**c**) Position on the 1RS chromosome arm of the markers studied [based on **Gyawali et al. (2010)**]. Polymorphic markers are enclosed by magenta rectangles. L: ladder; C: centromere


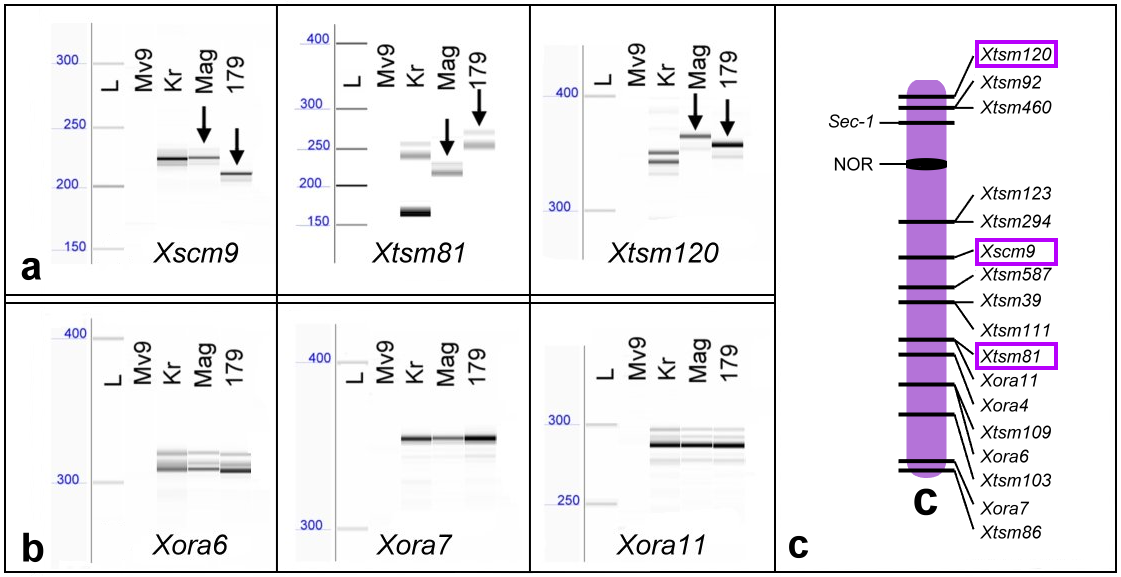


**Supplementary Tables**

**1RS arm of *Secale cereanum* ‘Kriszta’ confers resistance to stripe rust, improved grain yield and high arabinoxylan content in wheat**

Éva Szakács^1,#^, Kitti Szőke-Pázsi^1,#^, Balázs Kalapos^1^, Annamária Schneider^2^, László Ivanizs^1^, Marianna Rakszegi^1^, Gyula Vida^1^, István Molnár^1,3,*^ and Márta Molnár-Láng^1^

^1^Agricultural Institute, Centre for Agricultural Research, Martonvásár, H-2462, Hungary

^2^National Food Chain Safety Office, Budapest, 1024 Hungary

^3^Institute of Experimental Botany, Center of the Region Haná for Biotechnological and Agricultural Research, Šlechtitelů 31, CZ-78371 Olomouc, Czech Republic

*corresponding author e-mail: molnar.istvan@agrar.mta.hu

^#^these authors contributed equally to this work

**Supplementary Table S1** Adult plant responses to stripe rust (*Puccinia striiformis* f. sp. *tritici)* infection of the parental Mv9kr1 (wheat) and *Secale cereanum* cv. Kriszta, the wheat-*S. cereanum* 1R disomic addition line (DA1R) and the wheat-*S. cereanum* genotypes ‘179’ ‘C5’ and ‘D5’containing T1BL.1RS translocations, and the wheat cultivar Mv Magdaléna containing T1BL.1RS translocation of ‘Petkus’ origin (Martonvásár, 2014-2016).

| Genotype | Infection severity^a^ | | |
| --- | --- | --- | --- |
|  | 2014 | 2015 | 2016 |
| Mv9kr1 | 100% | 90% | 100% |
| Kriszta | 0 | 0 | 0 |
| Mv9kr1/Kriszta DA1R | 80% | 80% | 80% |
| Mv9kr1/Kriszta 1BL.1RS line ‘179’ | 0 | 0 | 0 |
| Mv9kr1/Kriszta 1BL.1RS line ‘C5’ | 0 | 0 | 0 |
| Mv9kr1/Kriszta 1BL.1RS line ‘D5’ | 0 | 0 | 0 |
| Mv Magdaléna | 80% | 60% | 70% |

^a^Infection severity was scored according to the modified Cobb scale (percentages of infected leaf area) **(Peterson et al. 1948)**.

**Supplementary Table S2** Virulence/avirulence formula of the pathotype population of leaf rust and the two patotype of powdery mildew used for artificial infection under greenhouse conditions.

| Disease | Isolate | Pathotype population | |
| --- | --- | --- | --- |
|  |  | virulent | avirulent |
|  |  | on differentials | |
| Leaf rust (*Puccinia triticina*) | - | *Lr1, 2a, 2b, 2c, 3, 3bg, 3ka, 10, 11, 12, 13, 14a, 14b, 15, 16, 17, 18, 20, 21, 22a, 22b, 23, 26, 30, 32, 33, 34, 35, 37, 38, 44, 51* | *Lr9, 19, 24, 25, 28, 29* |
|  |  |  |  |
| Powdery mildew (*Blumeria graminis f.sp. tritici*) | LH07-14 | *Pm1, 2, 3a, 3b, 3c, 3d, 4a, 4b, 5, 6, 7, 8, 17, 2+Mld, 2+6, 2+4b+8, 1+2+9* | *Pm3f* |
|  |  |  |  |
|  | LH14-14 | *Pm1, 2, 3c, 4a, 4b, 6, 7, 8, 17, 2+Mld, 2+6, 2+4b+8, 1+2+9* | *Pm3a, 3b, 3d, 3f, 5* |
|  |  |  |  |

**Supplementary Table S3** Seedling infection types produced by the parental wheat (Mv9kr1) and *Secale cereanum* (Kriszta) genotypes, the wheat-*S. cereanum* T1BL.1RS translocation ‘179’ and the wheat cultivar Mv Magdaléna containing T1BL.1RS translocation of ‘Petkus’ origin against leaf rust (*Puccinia triticina*) and powdery mildew (*Erysiphe graminis* f. sp. *tritici*).

| Genotype | Infection response | | |
| --- | --- | --- | --- |
|  | Leaf rust^a^ | Powdery mildew^b^ | |
|  |  | LH07-14 | LH14-14 |
| Wheat Mv9kr1 | 4 | 4 | 4 |
| Rye cv Kriszta | 0 | 0 | 0 |
| Wheat T1BL.1RS line ‘179’ | 0; | 4 | 4 |
| Wheat T1BL.1RS ‘Mv Magdalena | 4 | 4 | 4 |

^a^Infection types scored according to **Nover (1958)**: 0 = immune, 0; = hypersensitive, 1 = very resistant, 2 = moderately resistant, 3 = moderately susceptible, 4 = susceptible.

^b^Infection types scored according to **Stakman et al. (1962)**: 0 = resistant, 1 = resistant, 2 = resistant,

3 = susceptible, 4 = susceptible.

**Supplementary Table S4** List of the 1RS-specific SSR and ISBP markers used in the present study and their main PCR products amplified from DNA templates of ‘Mv Magdaléna’ and line ‘179’. Ta: annealing temperature, *:ISBP marker, **: polymorphic PCR product, ^#^: smeared band

| Marker | Ta (^o^C) | Amplicon size (bp) | | Marker | Ta (^o^C) | Amplicon size (bp) | |
| --- | --- | --- | --- | --- | --- | --- | --- |
|  |  | ‘Mv Magdaléna’ (T1BL.1RS) | ‘179’ (T1BL.1RS) |  |  | ‘Mv Magdaléna’ (T1BL.1RS) | ‘179’ (T1BL.1RS) |
| *Xscm*9^a^ | 60 | 224 | 211** | *Xtsm*123^b^ | 60 | 236 | 235 |
| *Xtsm*81^b^ | 60 | 216 | 255** | *Xtsm*294^b^ | 55 | 134, 131 | 135, 132 |
| *Xtsm*120^b^ | 60 | 365 | 357** | *Xtsm*460^b^ | 55 | 147 | 147 |
| *Xtsm*39^b^ | 60 | 195 | 194 | *Xtsm*587^b^ | 60 | 186, 174 | 188, 175 |
| *Xtsm*86^b^ | 55 | 210 | 211 | *Xora*2*^c^ | 62 | 355, 352 | 355, 352 |
| *Xtsm*92^b^ | 55 | 154 | 155 | *Xora*4*^c^ | 62 | 298, 295 | 299, 295 |
| *Xtsm*103^b^ | 60 | 190, 181 | 190, 180 | *Xora*6*^c^ | 62 | 321, 313, 309 | 320, 312, 308 |
| *Xtsm*109^b^ | 55 | 220, 217 | 219, 216 | *Xora*7*^c^ | 62 | 355 | 355 |
| *Xtsm* 111^b^ | 55 | 271-243^#^ | 270-242^#^ | *Xora*11*^c^ | 62 | 287 | 287 |

^a^**Saal and Wricke (1999)**, ^b^**Kofler et al. (2008)**, ^c^**Bartoš et al. (2008)**

**References**

**Kozub, N. A. *et al.*** Studying recombination between the 1RS arms from the rye Petkus and Insave involved in the 1BL.1RS and 1AL.1RS translocations using storage protein loci as genetic markers. *Cytol. Genet.* **52,** 440–447 (2018).

Gyawali, Y. P., Nasuda, S. & Endo, T. R. A cytological map of the short arm of rye chromosome 1R constructed with 1R dissection Stocks of Common Wheat and PCR-Based Markers. Cytogenet. Genome Res. 129, 224–233 (2010).

Peterson, R. F., Campbell, A. B. & Hannah, A. E. A diagrammatic scale for estimating rust intensity on leaves and stems of cereals. Can. J. Res. 26c, 496–500 (1948).

Nover, I. Sechsjährige Beobachtungen uber die physiologische Spezialisierung des echten Mehltaues (Erysiphe graminis DC) von Weizen und Gerste in Deutschland. J. Phytopathol. 31, 85–107 (1958).

Stakman, E., Steward, D. M. & Loegering, W. Q. Identification of physiologic races of Puccinia graminis var. tritici. USDA ARS E-617. US Gov. Print. Off., Washington, DC (1962).

Saal, B. & Wricke, G. Development of simple sequence repeat markers in rye (Secale cereale L.). Genome 42, 964–972 (1999).

Kofler, R. et al. Development of microsatellite markers specific for the short arm of rye (Secale cereale L.) chromosome 1. Theor. Appl. Genet. 117, 915–926 (2008).

**Bartoš, J. et al**. A first survey of the rye (Secale cereale) genome composition through BAC end sequencing of the short arm of chromosome 1R. BMC Plant Biology 8, 95 (2008).
